# Hyperuricemia: An Early Marker for Severity of Illness in Sepsis

**DOI:** 10.1155/2015/301021

**Published:** 2015-07-29

**Authors:** Sana R. Akbar, Dustin M. Long, Kashif Hussain, Ahmad Alhajhusain, Umair S. Ahmed, Hafiz I. Iqbal, Ailia W. Ali, Rachel Leonard, Cheryl Dalton

**Affiliations:** ^1^Division of Nephrology, Department of Medicine, West Virginia University School of Medicine, Morgantown, WV, USA; ^2^Division of Biostatistics, West Virginia University School of Medicine, Morgantown, WV, USA; ^3^Division of Pulmonary and Critical Care Medicine, Department of Medicine, West Virginia University School of Medicine, Morgantown, WV, USA; ^4^Department of Medicine, West Virginia University School of Medicine, Morgantown, WV, USA

## Abstract

*Background.* Uric acid can acutely activate various inflammatory transcription factors. Since high levels of oxyradicals and lower antioxidant levels in septic patients are believed to result in multiorgan failure, uric acid levels could be used as a marker of oxidative stress and poor prognosis in patients with sepsis. *Design.* We conducted a prospective cohort study on Medical Intensive Care Unit (MICU) patients and hypothesized that elevated uric acid in patients with sepsis is predictive of greater morbidity. The primary end point was the correlation between hyperuricemia and the morbidity rate. Secondary end points were Acute Kidney Injury (AKI), mortality, Acute Respiratory Distress Syndrome (ARDS), and duration of stay. *Results.* We enrolled 144 patients. 54 (37.5%) had the primary end point of hyperuricemia. The overall morbidity rate was 85.2%. The probability of having hyperuricemia along with AKI was 68.5% and without AKI was 31.5%. Meanwhile the probability of having a uric acid value <7 mg/dL along with AKI was 18.9% and without AKI was 81.1% (*p* value < 0.0001). *Conclusion.* We report that elevated uric acid levels on arrival to the MICU in patients with sepsis are associated with poor prognosis. These patients are at an increased risk for AKI and ARDS.

## 1. Introduction

In humans uric acid is the final oxidative product of purine metabolism through the action of xanthine oxidase or xanthine dehydrogenase. Approximately two-thirds of uric acid is excreted by the kidney, and the rest is excreted by the gastrointestinal tract. In addition some uric acid is degraded in the body after reaction with oxidants or peroxynitrite [1]. Uric acid occurs predominantly as a urate anion under physiologic pH. In the kidney, urate is filtered readily by the glomerulus and subsequently reabsorbed by the proximal tubular cells of the kidney; normal fractional excretion of uric acid is approximately 10% [2]. Normal levels of blood uric acid are typically 3.4–7.2 mg/dL for men and 2.4–6.1 mg/dL for women. Since the last century elevated uric acid levels have been noted to be associated with atherosclerosis [3–7], hypertension, hyperinsulinemia [8, 9], and chronic kidney disease [10]. Uric acid has been shown to be elevated in hypoxic states such as chronic heart failure [11, 12] and obstructive pulmonary disease [13, 14]. Hyperuricemia is defined as the accumulation of serum uric acid beyond its solubility point in water and develops due to uric acid overproduction, undersecretion, or both [15].

Uric acid can induce acute inflammation of the renal epithelial cells via uric acid crystals. Uric acid can also have an impact in the human body by its noncrystal effects. It may cause endothelial dysfunction and cause an afferent renal arteriolopathy and tubulointerstitial fibrosis in the kidney by activating the renin-angiotensin-aldosterone system [16], activate various inflammatory transcription factors [17], and induce systemic cytokine production such as tumor necrosis factor alpha [18] and local expression of chemokines such as monocyte chemotactic protein 1 in the kidney and cyclooxygenase 2 (COX-2) in blood vessels [19]. Experimentally induced hyperuricemia in rats leads to reduced urinary nitrite levels and systemic and glomerular hypertension [20, 21]. Other in vitro experimental studies have shown that uric acid decreases nitric oxide production [22] and also may lead to nitric oxide depletion [23]. The noncrystal effects of uric acid remain contentious because, under physiologic concentrations, urate is a powerful antioxidant that can scavenge superoxide, hydroxyl radicals, and singlet oxygen [24].

Sepsis is a serious medical condition characterized by a whole-body inflammatory state (systemic inflammatory response syndrome) and the presence of a known or suspected infection that has severe consequences [25]. Hence majority of intensive care unit patients undergo ischemic-reperfusion injury and inflammation to varying degrees during their hospitalization. Uric acid may be a factor playing a role in these processes since it has both oxidant and antioxidant properties. Since high levels of oxyradicals and lower antioxidant levels in patients with sepsis are believed to result in multiorgan failure, the measurement of uric acid levels could be possibly used as a marker of oxidative stress in patients with sepsis. Hence we decided to conduct a prospective cohort study in Medical Intensive Care Unit (MICU) patients admitted with sepsis to see if there is any significance of serum uric acid with respect to the morbidity rate. We hypothesized that elevated uric acid levels at the early hours of sepsis can predict an increased risk of morbidities as a single test.

## 2. Materials and Methods

### 2.1. Study Design

We conducted a prospective cohort study among patients admitted to the Medical Intensive Care Unit (MICU) at Ruby Memorial Hospital, West Virginia University (Morgantown, West Virginia), between January 2014 and July 2014. Patients or their Medical Power of Attorneys provided written informed consent and all the protocol was approved by the West Virginia University Office of Research Integrity and Compliance (West Virginia University Institutional Review Board). Funding for this study was provided through the West Virginia Clinical and Translational Science Institute Pilot Grants Program.

### 2.2. Enrollment Criteria

Inclusion criteria were age >18 years and admission to the MICU with a working diagnosis of sepsis based on the Society of Critical Care Medicine, Surviving Sepsis Campaign 2012 definition [26]. Exclusion criteria were as follows: (1) pregnant females and (2) patients from an outside facility that have already been in the MICU for more than 24 hours.

### 2.3. Data Collection and Definitions

Patients being admitted to the MICU were screened for sepsis. Sepsis was defined based on the Society of Critical Care Medicine, Surviving Sepsis Campaign 2012 definition [26]. Once the patient met the inclusion criteria then blood samples were obtained for uric acid, basic metabolic profile, complete blood count, lactic acid, phosphorus, albumin, and arterial blood gas. Repeat samples for arterial blood gas and basic metabolic profile were obtained at 24 and 48 hours. Subsequently, the electronic health records were reviewed to gather the remaining data such as the patients' age, sex, weight, race, body mass index, vital signs, comorbidities, ventilation status, need for renal replacement therapy, and hospital course over a 72-hour period. During the course of the study all patients continued to receive standard of care for their illnesses by the MICU team. For the purpose of our study we defined hyperuricemia as a uric acid level ≥7 mg/dL in both males and females. We defined Acute Kidney Injury (AKI) as an absolute ≥0.3 mg/dL increase in serum creatinine over a 48-hour time period from the baseline creatinine based on the Acute Kidney Injury Network (AKIN) definition [27]. We used as the baseline creatinine value the patients' creatinine value at the time of initial presentation to the MICU. We calculated the Acute Physiology and Chronic Health Evaluation (APACHE) II score based on Knaus et al. [28] definition to help assess the severity of disease in the MICU patient population. Acute Respiratory Distress Syndrome (ARDS) was defined per the Berlin definition [29].

### 2.4. Clinical Outcomes

The primary end point was the correlation between hyperuricemia in patients presenting with sepsis and the morbidity rate. We hypothesized that elevated uric acid in patients presenting to the MICU with sepsis is predictive of a greater morbidity rate. Hyperuricemia in general is defined as a serum urate level of >7 mg/dL (420 uM) in men and >6 mg/dL (300 uM) in women. For the purpose of our study we defined hyperuricemia as a uric acid level ≥7 mg/dL in both males and females. Secondary end points were Acute Kidney Injury (AKI), mortality, Acute Respiratory Distress Syndrome (ARDS), and duration of stay in MICU. AKI was defined as an absolute ≥0.3 mg/dL increase in serum creatinine over a 48-hour time period from the baseline creatinine based on the Acute Kidney Injury Network (AKIN) definition. We used as the baseline creatinine value the patients' creatinine value at the time of initial presentation to the MICU. Additional end points included need for renal replacement therapy and the patients' stability to be transferred to a lower level of care.

### 2.5. Statistical Analyses

Percentages of measures by uric acid level were compared using Chi squared tests for association. For APACHE II scores, linear regression was performed to assess the linear association with uric acid. All analyses were performed in SAS 9.4.

## 3. Results

### 3.1. Baseline Characteristics

**Figure 1:**
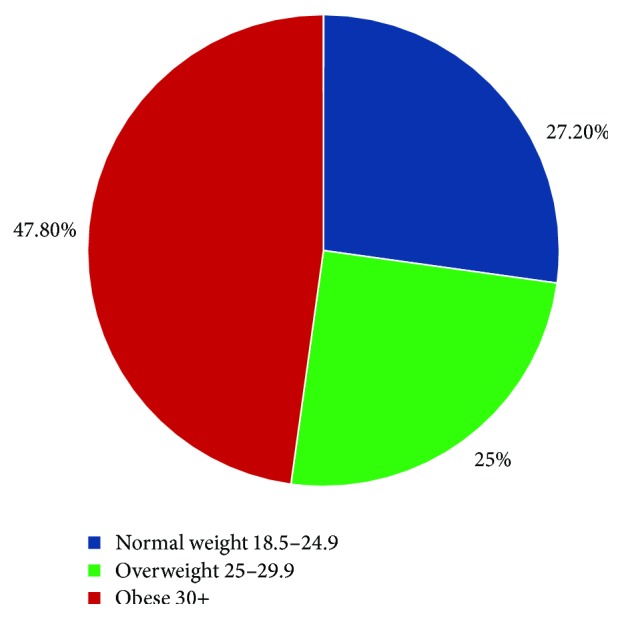
BMI distribution of the total patient population.

We enrolled and collected samples from 144 patients. The median age was 60.5 years. The most prevalent comorbidities were Diabetes, coronary artery disease, and cerebrovascular accident. Overall there were 57.6% males and 42.4% females and 39.6% of the enrolled patient population were ≥65 years of age. Our patient population was predominantly Caucasian (97.2%) (see Table 1). Also to note was that 47.8% of the overall patient population had a body mass index (BMI) ≥30 (see Table 1 and Figure 1). The BMI distribution in the overall population is given in Figure 1.

### 3.2. Acute Kidney Injury and Acute Respiratory Distress Syndrome

Amongst 144 patients, 54 (37.5%) had the primary end point of hyperuricemia. Within this subset of patients the overall morbidity rate was 85.2% of which AKI accounted for 68.5% and 83.8% had ARDS. Of those with ARDS 70.6% required mechanical ventilation. In terms of ARDS, although a high percentage of patients incurred this in our sample population, the incidence was not statistically significant most likely due to the small sample size of the study population. The probability of having hyperuricemia along with AKI is about 68.5% and without AKI is about 31.5%. Meanwhile the probability of having a uric acid value <7 mg/dL along with AKI is 18.9% and without AKI about 81.1%. These probabilities are statistically significant with a *p* value of <0.0001. Of the 37 patients who had hyperuricemia and AKI, only 3 (8.1%) needed renal replacement therapy; meanwhile for the overall sample 2.08% ended up having renal replacement therapy in the first 48 hrs of their MICU hospitalization.

**Figure 2:**
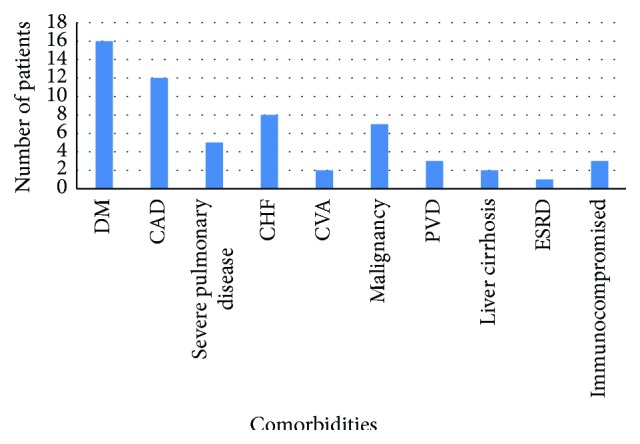
Comorbidities in patients with hyperuricemia and AKI.

The most prevalent comorbidities among patients with hyperuricemia were Diabetes Mellitus (40.7%), coronary artery disease (27.8%), and history of a malignancy (18.5%). Meanwhile the most prevalent comorbidities in patients with hyperuricemia who incurred AKI were Diabetes (43.3%), coronary artery disease (32.4%), and Congestive Heart Failure (21.6%) (see Figure 2).

### 3.3. Severity of Illness

Elevated lactic acid levels can be used as a marker for indicating impaired tissue oxygenation, leading to increased anaerobic metabolism and suggesting the presence of hemodynamic instability resulting in lack of appropriate organ perfusion. In our lab elevated lactic acid level was considered as any level >2.2 mmol/L. Of the patients with hyperuricemia 35.2% had an elevated lactic acid level. Our data is suggestive of an association between high uric acid levels and elevated lactic acid levels; however the results are not statistically significant most likely due to the small sample size.

**Figure 3:**
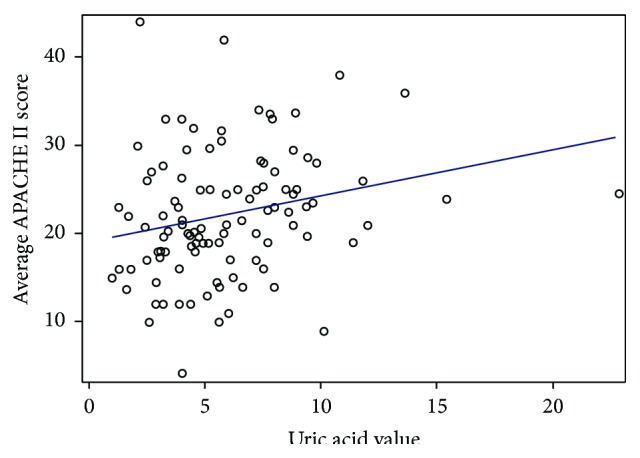
Uric acid levels and APACHE II score.

The Acute Physiology and Chronic Health Evaluation (APACHE) II score helps predict the severity of disease and the prognosis of the patients in the intensive care unit. For the purpose of our study we defined an elevated APACHE II score as any value ≥20 as scores of ≥20 have been associated with a greater than 35% predictive mortality rate. Of the 144 study patients 40 patients were excluded while calculating the APACHE II score as they did not have an arterial blood gas result. In our study of the patients with hyperuricemia 83.3% had an APACHE II score ≥20 while only 16.7% had an APACHE II score <20. The probability of having a uric acid level <7 mg/dL with an APACHE II score of ≥20 was 54.4% and with an APACHE II score of <20 was 45.6%. These probabilities are statistically significant with a *p* value of 0.0034. In addition a linear correlation between the APACHE II score and uric acid value was noted, *p* value of 0.014 (see Figure 3).

Hypoalbuminemia is a common problem associated with patients with acute and chronic medical conditions. The normal albumin values are 3.5–4.5 g/dL. We defined hypoalbuminemia as levels ≤3.5 gm/dL. Based on this of the patients with hyperuricemia 88.5% had hypoalbuminemia while 91.7% of those with a uric acid level <7 mg/dL had a low albumin level. The results were not statistically significant as the *p* value was 0.5367.

Hypophosphatemia has been hypothesized to be associated with early sepsis and the presence of elevated inflammatory cytokines. As per our results the overall incidence of hypophosphatemia in our sample population was only 16.7% and in the subgroup with hyperuricemia 7.4% had a serum phosphorus level of ≤2.5 mg/dL. Although of the nonhyperuricemic patients 22.2% had a low phosphorus level and these values are significantly different with *p* value of 0.0209, no correlation between hypophosphatemia and elevated uric acid levels was noted in our study.

Duration of stay in the MICU helps indirectly identify the degree of severity of illness of the ICU patients. We found that overall 75% and 54.2% of our enrolled patients were still in the MICU and not transferred to a lower level of care at 48 and 72 hours, respectively. The probability of having hyperuricemia and still being in the MICU at 48 and 72 hours was 81.5% and 64.8%, respectively, while the probability of having a uric acid level <7 mg/dL and being in the MICU at 48 and 72 hours was 71.1% and 47.8%, respectively. These probabilities are different with a *p* value of 0.1209 and 0.0464, respectively. For those with AKI, there was no difference in still being in the MICU by uric acid level, 82.4% versus 86.5% at 48 hours and 70.6% versus 70.6% at 72 hours. While not significant, there was a higher probability of still being in the MICU for those with ARDS and high uric acid levels compared to those with ARDS and low uric acid levels, 93.6% versus 81.5% (*p* value = 0.20) at 48 hours and 77.4% versus 68.5% (*p* value = 0.38).

## 4. Discussion

In this prospective cohort study, we report that elevated uric acid levels on arrival to the MICU in patients with sepsis are associated with a poor prognosis; that is, an increased risk for AKI, ARDS, marks an increased severity of illness measured as per the APACHE II score and increased duration of stay in the MICU. One may postulate that during sepsis there is an increased level of antioxidant response to counterbalance the excessive proinflammatory cytokines and oxidative stress, and this altered level of antioxidant defense leads to immune dysfunction and poor outcomes. In a systemic inflammatory response, both endothelial cells and neutrophils are activated to release oxygen-derived free radicals [30]. It seems that these oxyradicals play a role in causing or propagating the systemic inflammatory response syndrome (SIRS) in life-threatening conditions and that the imbalance in redox state reflects both oxidative stress and tissue damage [31, 32]. Serum uric acid, like other antioxidants such as albumin, bilirubin, or vitamins A, C, and E, is a powerful free radical scavenger and increases in response to acute oxidative stress [33, 34]. Uric acid formation may even provide a significant antioxidant defense mechanism against nitration by peroxynitrite in rat heart during hypoxia [35]. Hence uric acid is believed to be an important marker of oxidative stress.

The mechanisms for increased uric acid in sepsis are unknown and could be due to increased production as well as decreased excretion. Severe sepsis and septic shock may induce ischemia or hypoxia in multiple organs, which further increases the change in xanthine/hypoxanthine to uric acid through activation of xanthine oxidase in microvascular endothelium [36, 37]. When uric acid accumulates in blood vessels and deposits on the endothelium of vessels, the release of vasorelaxation factors is hampered [3], and vascular contraction interfered, leading to a series of pathophysiological processes and dysfunction of internal organs especially the kidney. Development of AKI during sepsis increases patient morbidity, predicts higher mortality, has a significant effect on multiple organ functions, is associated with an increased length of stay in the intensive care unit, and hence consumes considerable healthcare resources [25].

The first important finding of our study is that hyperuricemia is associated with AKI in patients with early sepsis. The development of AKI has significant effect on prognosis. For example, whereas the acute operative and postoperative mortality rate after cardiovascular surgery varies between 1 and 2%, this increases to 10 to 38% if renal insufficiency occurs and to >50% if dialysis is required [38, 39]. In general the septic patient population is a very complex subset of patients and majority of the time they have multiple organ involvement and are very sick patients. They are at risk for developing AKI due to changes in hemodynamics, exposure to various medications, changes in the functional capacity of other organs such as the heart and liver, and numerous other factors. Uric acid may be one of the factors contributing to it too.

Uric acid can cause AKI due to several mechanisms ranging from direct tubular toxicity from crystal induced injury to indirect injury secondary to the release of vasoactive mediators and oxidative stress. Uric acid can cause AKI secondary to renal vasoconstriction which occurs in response to the activation of the renin-angiotensin system, catecholamine release, oxidative stress, release of proinflammatory markers, and decreased nitric oxide levels. Renal vasoconstriction occurs in rats with experimentally induced hyperuricemia and is characterized by a marked increase in resistance of the afferent (and, to a lesser extent, efferent) arterioles and a reduction in single nephron GFR [40]. Uric acid strongly inhibits nitric oxide release from endothelial cells [41]. Khosla et al. [41] have demonstrated a reduction in plasma nitrites (metabolites of NO) in hyperuricemic rats that can be rescued by allopurinol. Uric acid levels have been shown by Zoccali et al. [42] to correlate with endothelial dysfunction [42]. Uric acid stimulates an inflammatory response via increasing various proinflammatory markers such as MCP and CRP. Hyperuricemic rats have a significant increase in macrophage infiltration in their kidneys independent of crystal deposition [20]. Despite having both oxidative and antioxidative properties, it appears that in periods of significant degrees of stress such as sepsis uric acids' protective antioxidative properties get overwhelmed and that despite increased levels of oxidative stress leading to increased uric acid levels the uric acid is more injurious than beneficial to the human body. Hence uric acid may be an early marker of impending AKI in patients with sepsis and could be used to predict the risk for AKI in septic patients. This further raises the question of whether the treatment of hyperuricemia in early sepsis could potentially decrease the risk for AKI.

Increased uric acid levels play a role not only in the occurrence of AKI but also in the progression of CKD. Uric acid levels are increased in subjects with renal disease as the result of reduction in GFR and renal urate excretion. Chonchol et al. have reported that uric acid levels are associated strongly with prevalent CKD [43]. Because of progressive loss of GFR, patients with CKD have decreased renal clearance of uric acid and thus greater serum uric acid levels than the general population [44].

The second important finding of our study is that hyperuricemia correlated with an elevated APACHE II score. This coincides with Jabs et al. [45] findings as they too found that plasma uric acid levels increased in relation to higher APACHE II scores. Chuang et al. [46] also found that an increase in serum uric acid had a positive correlation with total antioxidant capacity and APACHE II scores in patients with severe sepsis and septic shock. This suggests that uric acid may be an important contributor to total antioxidant capacity and that hyperuricemia may be an early predictive marker of poorer clinical outcomes in patients with sepsis. This raises the question then in the management of patients with early sepsis if the hyperuricemia should be treated to decrease the degree of injury that it may cause and decrease the morbidity and mortality rate in this patient population. Although it is known that uric acid has both oxidant and antioxidant properties the overall impact on the human body appears to be more injurious than protective. Hence perhaps the MICU can use uric acid level as a single marker to predict the severity of illness in critically ill patients presenting to the MICU rather than the slew of tests and variables needed to calculate the APACHE II score.

The third important finding of our study was that hyperuricemia noted in the septic population correlated with an increased probability of having the patient still in the MICU at 72 hours. This again suggests that uric acid may indeed be considered as a marker of severity of illness like the APACHE II score and can help predict that those with an elevated uric acid level at initial presentation are more likely to be still in the MICU at 72 hours versus those with a uric acid level less than 7 mg/dL. Thus raising thoughts that will treatment of hyperuricemia improve patient outcomes and decrease length of stay in the MICU?

In our study we found that although there was a high incidence of ARDS noted in this septic patient population, there was no statistically significant association of hyperuricemia with ARDS. Thus although uric acid levels may be used to predict severity of illness, length of stay in the MICU, and risk for AKI, it cannot at least at this time help us predict the incidence of ARDS. This could potentially be due to the small patient population that we had for our study, especially since increasing uric acid levels have been reported by Nagaya et al. [47] to correlate with clinical severity of primary pulmonary hypertension and has an independent association with long-term mortality of patient with primary pulmonary hypertension. Also from our study we found that although a high percentage of our patient populations were obese and a high percentage of those with hyperuricemia were obese, the results were not statistically significant. This was most likely due to the small sample size.

We acknowledge several limitations of this study, including modest sample size, enrollment of patients from the MICU and not the surgical intensive care unit, predominant Caucasian patient population, and short follow-up period. By virtue of the single-center study design, the results may not be generalizable to other MICU settings. If we had a larger sample size, perhaps the linear correlation between hyperuricemia with ARDS, obesity, and lactic acid levels may have become more apparent. Given the fact that the study center was located in West Virginia which is ranked as number two in terms of the state with the highest obesity rate in the USA, there could be potentially some degree of effect on our study results as hyperuricemia has been believed to often precede the development of obesity. So the possibility that our patient population already had an elevated uric acid level prior to being septic may be a factor increasing their risk of overall morbidity and degree of illness severity. Hence possibly we should test and potentially treat the general population for hyperuricemia to improve patient outcomes, which is a thought. Also to note is that our patient population was predominantly Caucasian, so the generalizability of the results is limited. The other limitations include that we did not have the baseline creatinine on the patients from prior to admission and hence did not know for certain what percentage of the patients had CKD prior to presentation. This is an important factor as patients with CKD are often noted to have an elevated uric acid level and it is debatable that elevated uric acid levels are injurious to the renal parenchyma as well as that CKD has been associated with decreased uric acid excretion and subsequently results in elevated uric acid levels. Thus, in order to minimize the degree of potential errors we used the patients' admission creatinine as the baseline value to mark the occurrence of AKI. Other potential limitations include the fact that we only followed the patients for 72 hours and that we did not trend the uric acid level during the course of the hospital stay to see if there was a potential change which was reflective of the patients hospital course. Throughout the analysis of the data we considered all the above-mentioned limitations especially the sample size and selection bias as it may have influenced our study results to a certain degree. Subsequently our study sets the stage for further randomized control trials that are multicenter and encompass a greater sample size and population diversity to help better elucidate and confirm our findings.

## 5. Conclusion

Our study findings simply demonstrate that hyperuricemia may be associated with poorer clinical outcomes in patients admitted to the MICU with sepsis. Serum uric acid levels may be potentially used as a sole marker of severity of illness as well as a predictor of morbidity in patients presenting to the MICU with sepsis. Further studies are needed to confirm our observations and elucidate the underlying mechanisms for hyperuricemia in sepsis.

## Figures and Tables

**Table 1:** Baseline characteristics.

Characteristics	Overall *N* (%)	Uric acid		*p* value^*∗*^
		High *N* (%)	Low *N* (%)	
Age				0.8519
<30 years old	10 (6.9%)	3 (5.6%)	7 (7.8%)	
30–65 years old	77 (53.5%)	30 (55.6%)	47 (52.2%)	
≥65 years old	57 (39.6%)	21 (38.9%)	36 (40.0%)	
Sex				0.9653
Females	61 (42.4%)	23 (42.6%)	38 (42.2%)	
Males	83 (57.6%)	31 (57.4%)	52 (57.8%)	
Ethnicity				0.2436
Caucasian	140 (97.2%)	51 (94.4%)	89 (98.9%)	
Black	3 (2.1%)	2 (3.7%)	1 (1.1%)	
BMI				0.0195
18.5–24.9	37 (27.2%)	12 (22.6%)	25 (30.1%)	
25–29.9	34 (25.0%)	8 (15.1%)	26 (31.3%)	
≥30	65 (47.8%)	33 (62.3%)	32 (38.6%)	
Comorbidities				
DM	53 (36.8%)	22 (40.7%)	31 (34.4%)	0.4482
CAD	37 (25.7%)	15 (27.8%)	22 (24.4%)	0.6576
Severe pulmonary disease	23 (16.0%)	7 (13.0%)	16 (17.8%)	0.4452
CHF	14 (9.7%)	8 (14.8%)	6 (6.7%)	0.1101
CVA	24 (16.7%)	7 (13.0%)	17 (19.9%)	0.3556
h/o malignancy	23 (16.0%)	10 (18.5%)	13 (14.4%)	0.5182

^*∗*^Chi square test.
